# Intervention Models in Functional Connectivity Identification Applied to fMRI

**DOI:** 10.1155/IJBI/2006/27483

**Published:** 2006-09-05

**Authors:** João Ricardo Sato, Daniel Yasumasa Takahashi, Ellison Fernando Cardoso, Maria da Graça Morais Martin, Edson Amaro Júnior, Pedro Alberto Morettin

**Affiliations:** ^1^ Departamento de Estatística, Instituto de Matemática e Estatística, Universidade de São Paulo, São Paulo, Sp 05508-090, Brazil; ^2^ Laboratório de Neuroimagem Funcional (NIF), Lim 44, Faculdade de Medicina, Universidade de São Paulo, São Paulo, Sp 05403-001, Brazil; ^3^ Departamento de Radiología, Hospital das Clínicas, Faculdade de Medicina, Universidade de São Paulo, São Paulo, Sp 05403-001, Brazil

## Abstract

Recent advances in neuroimaging techniques have provided precise spatial localization of brain activation applied in several neuroscience subareas. The development of functional magnetic resonance imaging (fMRI), based on the BOLD signal, is one of the most popular techniques related to the detection of neuronal activation. However, understanding the interactions between several neuronal modules is also an important task, providing a better comprehension about brain dynamics. Nevertheless, most connectivity studies in fMRI are based on a simple correlation analysis, which is only an association measure and does not provide the direction of information flow between brain areas. Other proposed methods like structural equation modeling (SEM) seem to be attractive alternatives. However, this approach assumes prior information about the causality direction and stationarity conditions, which may not be satisfied in fMRI experiments. Generally, the fMRI experiments are related to an activation task; hence, the stimulus conditions should also be included in the model. In this paper, we suggest an intervention analysis, which includes stimulus condition, allowing a nonstationary modeling. Furthermore, an illustrative application to real fMRI dataset from a simple motor task is presented.


Hindawi Publishing Corporation
International Journal of Biomedical Imaging
Volume 2006, Article ID 27483, Pages 1–7
DOI 10.1155/IJBI/2006/27483

### Intervention Models in Functional Connectivity Identification Applied to fMRI

**João Ricardo Sato,[1,2] Daniel Yasumasa Takahashi,[3] Ellison Fernando Cardoso,[2,3] Maria da Graça Morais Martin,[2,3] Edson Amaro Júnior,[2,3] and Pedro Alberto Morettin[1]**

[1] *Departamento de Estatística, Instituto de Matemática e Estatística, Universidade de São Paulo, São Paulo, Sp 05508-090, Brazil*
[2] *Laboratório de Neuroimagem Funcional (NIF), Lim 44, Faculdade de Medicina, Universidade de São Paulo, São Paulo, Sp 05403-001, Brazil*
[3] *Departamento de Radiología, Hospital das Clínicas, Faculdade de Medicina, Universidade de São Paulo, São Paulo, Sp 05403-001, Brazil*

Received 31 January 2006; Revised 26 June 2006; Accepted 26 June 2006

Recent advances in neuroimaging techniques have provided precise spatial localization of brain activation applied in several neuroscience subareas. The development of functional magnetic resonance imaging (fMRI), based on the BOLD signal, is one of the most popular techniques related to the detection of neuronal activation. However, understanding the interactions between several neuronal modules is also an important task, providing a better comprehension about brain dynamics. Nevertheless, most connectivity studies in fMRI are based on a simple correlation analysis, which is only an association measure and does not provide the direction of information flow between brain areas. Other proposed methods like structural equation modeling (SEM) seem to be attractive alternatives. However, this approach assumes prior information about the causality direction and stationarity conditions, which may not be satisfied in fMRI experiments. Generally, the fMRI experiments are related to an activation task; hence, the stimulus conditions should also be included in the model. In this paper, we suggest an intervention analysis, which includes stimulus condition, allowing a nonstationary modeling. Furthermore, an illustrative application to real fMRI dataset from a simple motor task is presented.

Copyright © 2006 João Ricardo Sato et al. This is an open access article distributed under the Creative Commons Attribution License, which permits unrestricted use, distribution, and reproduction in any medium, provided the original work is properly cited.

### 1. INTRODUCTION

Functional magnetic resonance imaging (fMRI) based on blood oxygenation level-dependent (BOLD) signal has become one of the most prominent and powerful tools in cognitive neuroscience [1]. Most fMRI studies found in the literature focus on the detection of neuronal activation and brain mapping via statistical analysis. However, understanding cortical dynamics is a crucial step toward inferring cortical functioning.

Several evidences [2–4] suggest that modeling the interactions between different brain structures is paramount to understand the mechanisms guiding specific cognitive behaviour. However, the determination of parameters involved in cortical dynamics is still an open question. A number of techniques are being used to detect patterns of interaction between cortical areas, most using an *ad hoc* concept. So far, most connectivity studies have investigated temporal correlation as a measure of connectivity [3], even though it is not enough to identify the direction of information flow. In fact, Pearson correlation coefficient in time series analysis is just a measure of linear association. The connectivity mapping via correlation analysis is obtained firstly by selecting a seed voxel, and then Pearson correlation is calculated against all the other brain voxels. In most cases, the selection of the seed voxel is derived from the activation maps. Hence, as the activation detection is based on the similarity between the observed BOLD signal in a voxel and an expected haemodynamic curve, the correlation connectivity analysis is close to an activation mapping considering the seed voxel BOLD signal as the expected curve. Finally, we conclude that the correlation connectivity mapping is not sufficient to provide additional information in relation to the activation analysis based on general linear model (GLM).

Other statistical methods, such as the structural equation modeling (SEM), are more attractive to overcome this shortcoming. Büchel and Friston [2] modeled the occipitoparietofrontal network involved in attention tasks using structural equation modeling. Zhuang et al. [5] applied SEM to a bimanual motor coordination experiment. Rowe et al. [6] modeled the prefrontal cortex in a color selection task. An improvement of SEM applied to fMRI analysis is the dynamic causal model (DCM), proposed by Friston et al. [7]. However, these two modeling approaches require a complete prespecification of the connectivity structure. Additionally, as DCM is estimated via Bayesian algorithms, it also requires the prior densities of the parameters of interest. In fact, these models measure the instantaneous connectivity, but require the direction of information flow. Therefore, these approaches are not enough to provide a complete identification of the connectivity pattern. Furthermore, the autocorrelation of BOLD signal is another obstacle for the application of these models and, in most cases, is simply ignored.

Granger causality [8] is a very prominent concept to describe information flow and connectivity. Baccalá et al. [9] and Baccalá and Sameshima [10] introduced a frequency-domain connectivity identification method for EEG using partial directed coherence and this causality concept. Goebel et al. [11] and Roebroeck et al. [12] introduced the concept of Granger causality in fMRI via vector autoregressive modeling (VAR). They have also shown the applicability of this approach to BOLD signals using simulations and illustrating it with real data derived from visuomotor studies. Compared to other approaches described previously, the main advantage of Granger causality identification via VAR models is the fact that prior specifications about connectivity structure are not necessary. Also, if one has prior partial knowledge about this structure, it can be naturally included in the model as a restriction in the parameters to be estimated.

The stationarity condition is one of the main obstacles to VAR modeling application in fMRI analysis. This assumption requires the connectivity structure to be the same during all acquisition times. Although acceptable in resting state or one-condition experiments, it may not be valid in paradigms with more than one condition. In that matter, as the connectivity structure may change according to the stimulus, comparisons between these structures are also an interesting point.

In this paper, we propose the use of a generalization of VAR models via intervention analysis (structural break models), which allows a natural modeling of connectivity and also statistical comparisons of the connectivity structures in experiments with different stimulus conditions.

### 2. GRANGER CAUSALITY AND CONNECTIVITY

In fMRI analysis, the definition of connectivity can be divided into two concepts: functional and effective connectivity. The first is defined as "*correlations between spatially remote neurophysiological events*" [4]. In contrast, effective connectivity is related to the "*influence of one neural system over another*" [4]. Note that effective connectivity implies in functional one, but the reciprocal may not be valid. The activity correlations or synchronisms may be observed due to external factors, not only due to synaptic interactions between the areas involved. An illustrative example involving differences between these two concepts of connectivity could be a paradigm with simultaneous stimulation of visual and auditory cortex. The neuronal activity in these areas will be correlated (functional), but not related to neural interactions (effective). However, the simplicity of functional connectivity concept makes it very useful, mainly in cases where the neural activity is measured indirectly, as in fMRI time series.

Granger causality is a very useful concept for the description of brain areas connectivity and direction of information flow identification [10, 13]. In the context of time series, Granger [8] defined causality in terms of predictability. This concept was originated in econometrics, focusing the understanding of relationships between financial time series such as prices, indexes, interest rates, and so forth. The basis of this concept is that effect cannot precede cause, suggesting that causality can be detected toward past and future relationships. A signal $x_t$ is said to Granger-cause a signal $y_t$ if the past values of $x_t$ help the prediction of present values of $y_t$. In other words, if the variance of the prediction error of $y_t$, considering all the information until the time $t$, is less than the one obtained excluding the information of past values of $x_t$, then $x_t$ is said to Granger-cause $y_t$.

Goebel et al. [11] introduced Granger causality identification between BOLD time series using VAR models, showing the applicability of this approach in simulated and real fMRI data. Note that Granger causality aims to identify interactions and relationships between signals via precedence and prediction. However, it is more related to functional connectivity than effective one, because it cannot distinguish real influences from prediction power.

Let a $k$-dimensional multivariate time series

$$\mathbf{Y}_t = \begin{bmatrix} y_{1t} & y_{2t} & \cdots & y_{kt} \end{bmatrix}', \quad t = 1, 2, \ldots, T, \tag{1}$$

composed by $k$ signals measured on time $t$. In order to measure the prediction improvement of $\mathbf{Y}_t$ using a collection of $p$ past values of the series ($\mathbf{Y}_{t-1}, \mathbf{Y}_{t-2}, \ldots, \mathbf{Y}_{t-p}$), assume a $k$-dimensional vector autoregressive model (VAR) of order $p$,

$$\mathbf{Y}_t = \mathbf{v} + \mathbf{A}_1\mathbf{Y}_{t-1} + \mathbf{A}_2\mathbf{Y}_{t-2} + \cdots + \mathbf{A}_p\mathbf{Y}_{t-p} + \mathbf{u}_t, \tag{2}$$

where $\mathbf{v}$ is the intercept vector (related to the process average), $\mathbf{u}_t$ is an error vector of random variables with zero mean and covariance matrix $\mathbf{\Sigma}$ given by

$$\mathbf{\Sigma} = \begin{bmatrix} \sigma_{11}^2 & \sigma_{12} & \cdots & \sigma_{k1} \\ \sigma_{21} & \sigma_{22}^2 & \cdots & \sigma_{k2} \\ \sigma_{31} & \sigma_{32} & \cdots & \sigma_{k3} \\ \vdots & \vdots & \ddots & \vdots \\ \sigma_{k1} & \sigma_{k2} & \cdots & \sigma_{kk}^2 \end{bmatrix}, \tag{3}$$

$\mathbf{v}$ and $\mathbf{A}_i$ are coefficient matrices given by

$$\mathbf{v} = \begin{bmatrix} v_1 \\ v_2 \\ \vdots \\ v_k \end{bmatrix}, \qquad \mathbf{A}_i = \begin{bmatrix} a_{11i} & a_{12i} & \cdots & a_{1ki} \\ a_{21i} & a_{22i} & \cdots & a_{2ki} \\ a_{31i} & a_{32i} & \cdots & a_{3ki} \\ \vdots & \vdots & \ddots & \vdots \\ a_{k1i} & a_{k2i} & \cdots & a_{kki} \end{bmatrix}, \quad i = 1, 2, \ldots, p. \tag{4}$$

As the error term $\mathbf{u}_t$ has zero mean, the predicted values of $\mathbf{Y}_t$ conditional to the past values are given by

$$\hat{\mathbf{Y}}_t = \mathbf{v} + \mathbf{A}_1\mathbf{Y}_{t-1} + \mathbf{A}_2\mathbf{Y}_{t-2} + \cdots + \mathbf{A}_p\mathbf{Y}_{t-p}. \tag{5}$$

The VAR model allows an easy way to identify Granger causality. If the coefficient $a_{jli}$ for some $i$ is nonzero, we say that signal $y_{lt}$ Granger-causes the signal $y_{jt}$. In other words, the past values of the signal $y_{lt}$ help the prediction of the present and future values of the signal $y_{jt}$. It is important to mention that this kind of relationship is not reciprocal, for example, $y_{lt}$ may Granger-cause the signal $y_{jt}$, but not necessarily $y_{jt}$ causes $y_{lt}$, indicating the direction of information flow.

Consider a functional magnetic resonance dataset. Select $k$ voxels in the volume, obtaining a BOLD $k$-dimensional signal. Using the concept of Granger causality and the VAR modeling, it is possible to verify if the BOLD signal of certain brain areas Granger-causes another areas' BOLD signal, by testing the significance of the estimates of matrix $\mathbf{A}_t$. Therefore, we are able to test the functional connectivity and direction of the information flow.

However, VAR modeling is only suitable in cases of stationary time series with coefficients and error covariance matrix invariant on time. Hence, considering fMRI studies, a weakness of this approach is the assumption that both activation and connectivity functions are constant in the whole scanning interval. Let an epoch functional magnetic resonance image experiment with two conditions $A$ and $B$. It is reasonable to expect functional connectivity under $A$, but it may not be the same under $B$. Therefore, we propose the use of intervention VAR (structural breaks model) focusing on the identification of changes in functional connectivity. The intervention VAR model is defined by

$$\mathbf{Y}_t = \mathbf{v}^{(C)} + \mathbf{A}_1^{(C)}\mathbf{Y}_{t-1} + \mathbf{A}_2^{(C)}\mathbf{Y}_{t-2} + \cdots + \mathbf{A}_k^{(C)}\mathbf{Y}_{t-k} + \mathbf{u}_t, \tag{6}$$

where the coefficient matrices are given by

$$\mathbf{v}^{(C)} = \begin{bmatrix} v_1 + \delta_1^{(C)} \\ v_2 + \delta_2^{(C)} \\ \vdots \\ v_k + \delta_k^{(C)} \end{bmatrix} = \mathbf{v} + \boldsymbol{\delta}^{(C)},$$

$$\mathbf{A}_i^{(C)} = \begin{bmatrix} a_{11i} + \partial_{11i}^{(C)} & a_{12i} + \partial_{12i}^{(C)} & \cdots & a_{1ki} + \partial_{1ki}^{(C)} \\ a_{21i} + \partial_{21i}^{(C)} & a_{22i} + \partial_{22i}^{(C)} & \cdots & a_{2ki} + \partial_{2ki}^{(C)} \\ a_{31i} + \partial_{31i}^{(C)} & a_{32i} + \partial_{32i}^{(C)} & \cdots & a_{3ki} + \partial_{3ki}^{(C)} \\ \vdots & \vdots & \ddots & \vdots \\ a_{k1i} + \partial_{k1i}^{(C)} & a_{k2i} + \partial_{k2i}^{(C)} & \cdots & a_{kki} + \partial_{kki}^{(C)} \end{bmatrix} = \mathbf{A}_i + \partial_i^{(C)}, \tag{7}$$

$\mathbf{u}_t$ is an error vector of random variables with zero mean and covariance matrix $\mathbf{\Sigma}^{(C)}$ defined by

$$\begin{aligned}\mathbf{\Sigma}^{(C)} &= \begin{bmatrix} \sigma_{11}^2 + \psi_{11}^{(C)} & \sigma_{12} + \psi_{12}^{(C)} & \cdots & \sigma_{1k} + \psi_{1k}^{(C)} \\ \sigma_{21} + \psi_{21}^{(C)} & \sigma_{22}^2 + \psi_{22}^{(C)} & \cdots & \sigma_{2k} + \psi_{2k}^{(C)} \\ \sigma_{31} + \psi_{31}^{(C)} & \sigma_{32} + \psi_{32}^{(C)} & \cdots & \sigma_{3k} + \psi_{3k}^{(C)} \\ \vdots & \vdots & \ddots & \vdots \\ \sigma_{k1} + \psi_{k1}^{(C)} & \sigma_{k2} + \psi_{k2}^{(C)} & \cdots & \sigma_{kk}^2 + \psi_{kk}^{(C)} \end{bmatrix} \\ &= \mathbf{\Sigma} + \Psi^{(C)},\end{aligned} \tag{8}$$

and $C$ indicates the block condition ($A$ or $B$). For simplicity, assume that

$$\delta_j^{(A)} = 0, \qquad \partial_{jli}^{(A)} = 0, \qquad \psi_{jl}^{(A)} = 0 \tag{9}$$

for $j = 1, 2, \ldots, k$, $l = 1, 2, \ldots, k$, $i = 1, 2, \ldots$, and $\delta_j^{(B)}$, $\partial_{jli}^{(B)}$, and $\psi_{jl}^{(B)}$ are the increments on the coefficients during $B$ condition. If at least one of the coefficients $\delta_j^{(B)}$, $\partial_{jli}^{(B)}$, and $\psi_{jl}^{(B)}$ is nonzero, it implies the existence of structural changes. In other words, we have different coefficient matrices for each condition. Thus, we have a VAR structure for each block, but all the parameters are globally estimated, allowing a statistical test to the connectivity changes. The intervention VAR model can be estimated using an interactive generalized least-square estimator. Consider the following vector and matrices:

$$\begin{aligned} \mathbf{y} &= \operatorname{vec}(\mathbf{Y}_t), \\ \mathbf{X} &= \begin{bmatrix} \mathbf{1} & \mathbf{Y}_{t-1} & \mathbf{Y}_{t-2} & \cdots & \mathbf{Y}_{t-p} \end{bmatrix}, \\ \mathbf{Z} &= \mathbf{I}_k \otimes ([\mathbf{1} \quad \Delta] \otimes^R \mathbf{X}), \end{aligned} \tag{10}$$

where vec is an operator that concatenates all the columns of a matrix in a column vector, $\Delta$ is a vector of zeros and ones indicating the stimulus condition at time $t$ (the $t$th element of $\Delta$ is zero/one if the acquisition at time $t$ occurs during $A/B$ condition), $\mathbf{1}$ is a column vector of ones, and $\otimes^R$ is the row-Kronecker product, which is defined as the Kronecker product applied separately for each row, that is,

$$\begin{bmatrix} a_1 \\ a_2 \\ \vdots \\ a_k \end{bmatrix} \otimes^R \begin{bmatrix} b_1 \\ b_2 \\ \vdots \\ b_k \end{bmatrix} = \begin{bmatrix} a_1 \otimes b_1 \\ a_2 \otimes b_2 \\ \vdots \\ a_k \otimes b_k \end{bmatrix}. \tag{11}$$

The error covariance matrix is given by $\mathbf{\Gamma}$. The generalized least-square estimator of the coefficients of the intervention VAR model is given by [14, 15]

$$\hat{\boldsymbol{\beta}} = \left(\mathbf{Z}'\mathbf{\Gamma}^{-1}\mathbf{Z}\right)^{-1}\mathbf{Z}'\mathbf{\Gamma}^{-1}\mathbf{y}. \tag{12}$$

However, as the covariance matrix $\mathbf{\Gamma}$ is unknown, we propose the use of an interactive two-stage least-square estimator. The residuals are estimated on the first step and the covariance matrix $\mathbf{\Gamma}$ on the second one, as an extension of the Cochrane-Orcutt procedure. The variances/covariances in $\mathbf{\Gamma}$

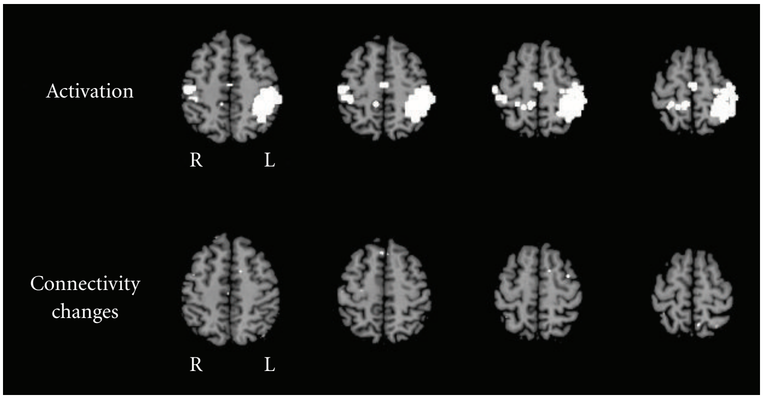

Figure 1: Group activation and connectivity changes maps for six subjects in a rest-fingertap block design paradigm. The connectivity changes map shows the voxels with significant changes in the information flow intensity from SMA, between rest and fingertap condition. The maps are presented on radiological convention.

may be consistently estimated considering the fits of an ordinary regression of squares/cross residuals as response and $[\mathbf{1}\ \ \Delta]$ as model matrix. The Wald test statistic of linear combinations of parameter in $\boldsymbol{\beta}$ is given by

$$W = \frac{(\mathbf{C}\widehat{\boldsymbol{\beta}} - \mathbf{m})'\left[\mathbf{C}(\mathbf{Z}'\boldsymbol{\Gamma}^{-1}\mathbf{Z})\mathbf{C}'\right]^{-1}(\mathbf{C}\widehat{\boldsymbol{\beta}} - \mathbf{m})}{\widehat{\sigma}^2}, \tag{13}$$

where $\widehat{\sigma}^2$ is the estimated residual variance, $\mathbf{m}$ is a vector, and $\mathbf{C}$ is a contrast matrix corresponding to the following test:

$$\begin{aligned} H_0 &: \mathbf{C}\boldsymbol{\beta} = \mathbf{m}, \\ H_A &: \mathbf{C}\boldsymbol{\beta} \neq \mathbf{m}. \end{aligned} \tag{14}$$

Under the null hypothesis, $W$ has an asymptotic chi-square distribution with *rank* ($\mathbf{C}$) degrees of freedom [14]. Basically, this procedure performs simultaneous tests of the equality between $\mathbf{m}$ and linear combinations ($\mathbf{C}$) of parameters in $\boldsymbol{\beta}$. Note that the connectivity parameters can be easily tested considering the $W$ statistic. Further, in case of non-Gaussian errors distribution, the martingales central limit theorem [16] implies that the classic asymptotic properties of the generalized least-square estimator are valid.

To finish this section, it is important to highlight some points about the application of the intervention VAR models to connectivity analysis in fMRI. Firstly, although Granger causality identification via VAR models is closely related to interactions, it cannot make a distinction between real influences or predictive power. Secondly, it is important to mention that this approach depends on sampling frequency. As Granger causality identification is based on information contained in past values, low sampling rates result in aliasing and data aggregation. In fact, sampling frequency represents a challenge for connectivity modeling in fMRI, as short acquisition time implies in low signal-to-noise ratio. On the other hand, neural interactions occur in a frequency much higher than fMRI acquisitions (TR). Thus, the connectivity structure identified via VAR models is only related to very low frequencies information, and fast interactions are not detected. Additionally, we would like to emphasize the point that although intervention model does not require the assumption of global stationarity, it depends on this assumption to be valid during each paradigm condition. The validity of this assumption is reasonable in block design paradigms, but it may not be true in event-related ones. The application of the proposed approach to fMRI data with event-related designs could result in an imprecise estimation, such as an average of the information flow intensity across the activation time-points.

### 3. APPLICATION TO REAL DATA

In order to illustrate the usefulness of the proposed approach, the intervention VAR modeling was applied to real fMRI data derived from a motor task study.

Six normal right-handed subjects performed a simple right hand fingertapping task, in an AB periodic block design experiment. The functional magnetic resonance images were acquired in a GE 1.5 T Signa LX MR system equipped with a 23 mT/m gradient, (TE: 40 milliseconds, TR: 2000 milliseconds, FA: 90°, FOV: 240 mm, 64 × 64 matrix; 15 slices, thickness: 7.0 mm, gap: 0.7 mm) oriented in the AC-PC plane in a single run. There were one hundred volumes collected during five cycles of rest-task performance. Each cycle had the duration of 40 seconds corresponding to 20 volumes, 10 volumes acquired during rest, and 10 volumes during the activation task. The subjects were in the dark room with noise-reducing headphones customized for functional MR. Instructions to begin and finish movements were given via auditory stimuli.

The images were preprocessed considering motion correction and spatial smoothing. The activation brain mapping was obtained using the XBAM software [17]. Spatial normalization transformation to the stereotatic space of Talairach and Tournoux [18] was performed using SPM2 [4]. Since the main interest was the study of motor function, the analyses were limited to the superior slices, reducing the number of multiple comparisons and consequently increasing the sensitivity of the statistical tests.

The group activation maps (cluster $P$ value $< .01$) are presented in Figure 1 (top). Note that there are significant activations found on the left primary and contralateral motor cortex (BA 4), supplementary and premotor areas (BA 6), and primary sensitive areas (BA 3, 2, 1), which are classically involved in motor control.

Taking into account a significant activation in the supplementary motor area (SMA), the intervention VAR analysis was performed in a bivariate fashion considering a seed in the activation local maxima of SMA against all other voxels in the whole brain. As changes in BOLD signal are not instantaneous, we considered a delay of 2 seconds in the condition specification. Statistical tests for differences in the information flow intensity from SMA to the voxel of interest, between rest and task, were performed in each individual separately. The individual $W$ statistics were mapped to Gaussian quantiles, and the group analysis was performed in SPM2 (on-tailed $t$ test, which is similar to a random effects analysis [4]). The connectivity changes map (voxel $P$ value $< .005$) is presented in Figure 1 (bottom). The areas with significant changes in the information flow intensity from SMA were the premotor cortex, presupplementary motor area, and primary sensitive cortex.

The intervention VAR model was also applied in a trivariate analysis, considering the selected seed in SMA, and voxels (connectivity changes minimum $P$ value) in the pre-motor cortex (PM) and pre-supplementary motor area (PSMA). In Figure 2 (top), diagrams (arrow $P$ value $< .05$) describing the connectivity structure during rest and fingertap and also differences in information flow intensity are presented. The BOLD signals derived from selected ROI of one subject are presented in Figure 2 (bottom).

When contrasting fingertapping with rest we found significant changes in PM cortex, primary sensitive areas, and PSMA. All these areas are classically involved in movement control [19]. The current understanding of motor control in the literature suggests that PSMA provides the main input to SMA, which is possibly responsible for providing internal representation of movement sequences, and is involved in learning process of new movements. SMA is believed to send information to the PM and primary motor cortices, and is also associated with complex calculation to achieve maximum performance, based on feedback information from sensitive areas.

During the rest, we observed increased connectivity between the SMA and PM with pre-SMA. Some studies demonstrated that PSMA plays an important role in cognitive motor control, which involves sensory discrimination and movement decision making (go/nongo) or motor selection for the action after stimuli [20, 21]. In order to start the sequence of fingertapping, a motor decision must be made, based on the instruction previously given to the participant. All areas (pre-SMA, PM, and SMA) are involved in the initiation of movement and modification in their connectivity pattern might be explained by the attention to stimulus presentation and monitoring during the task.

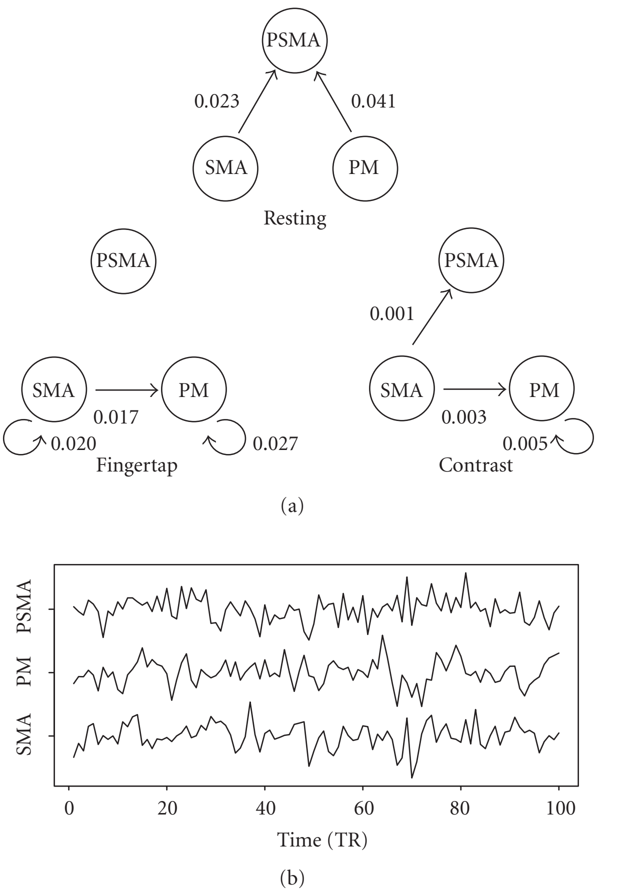

Figure 2: Group connectivity structure during resting, fingertapping, and information flow changes between these two conditions (top). The numbers in the arrows describe the information flow correspondent $P$ value. An illustrative chart of ROI's BOLD signals of one subject is also presented.

### 4. CONCLUSION

Most connectivity studies results rely on analyzing second-order correlations that do not give additional information about neural interactions. Other advanced methods like structural equation modeling (SEM) or dynamic causal models (DCM) could be an attractive alternative. However, they heavily depend on a prior knowledge about involved neural circuitry.

Granger causality concept, in its most general form, is a flexible definition of relationship and temporal order. It could be tested by a simple VAR modeling without any connectivity prespecification. In this paper, we introduced a new approach for connectivity modeling based on Granger causality and intervention VAR models, which enabled us to compare differences in connectivity structures as a statistical hypothesis test. An initial application of intervention VAR models to fMRI data produced biologically plausible results, but further experiments are necessary to reveal its potential as a new tool to investigate neural systems.

### ACKNOWLEDGMENT

This research was supported by FAPESP (03/10105-2) and CNPq (142616/2005-2), Brazil.

### REFERENCES

[1] S. Ogawa, T.-M. Lee, A. S. Nayak, and P. Glynn, "Oxygenation-sensitive contrast in magnetic resonance image of rodent brain at high magnetic fields," *Magnetic Resonance in Medicine*, vol. 14, no. 1, pp. 68–78, 1990.

[2] C. Büchel and K. J. Friston, "Modulation of connectivity in visual pathways by attention: cortical interactions evaluated with structural equation modelling and fMRI," *Cerebral Cortex*, vol. 7, no. 8, pp. 768–778, 1997.

[3] B. Biswal, F. Z. Yetkin, V. M. Haughton, and J. S. Hyde, "Functional connectivity in the motor cortex of resting human brain using echo-planar MRI," *Magnetic Resonance in Medicine*, vol. 34, no. 4, pp. 537–541, 1995.

[4] R. S. J. Frackowiak, K. J. Friston, C. Frith, et al., *Human Brain Function*, Academic Press, San Diego, Calif, USA, 2nd edition, 2003.

[5] J. Zhuang, S. LaConte, S. Peltier, K. Zhang, and X. Hu, "Connectivity exploration with structural equation modeling: an fMRI study of bimanual motor coordination," *NeuroImage*, vol. 25, no. 2, pp. 462–470, 2005.

[6] J. B. Rowe, K. E. Stephan, K. Friston, R. S. J. Frackowiak, and R. E. Passingham, "The prefrontal cortex shows context-specific changes in effective connectivity to motor or visual cortex during the selection of action or colour," *Cerebral Cortex*, vol. 15, no. 1, pp. 85–95, 2005.

[7] K. J. Friston, J. Ashburner, C. D. Frith, J.-B. Poline, J. D. Heather, and R. S. J. Frackowiak, "Spatial registration and normalization of images," *Human Brain Mapping*, vol. 3, no. 3, pp. 165–189, 1995.

[8] C. W. J. Granger, "Investigating causal relations by econometric models and cross-spectral methods," *Econometrica*, vol. 37, no. 3, pp. 424–438, 1969.

[9] L. A. Baccalá, K. Sameshima, G. Ballester, A. C. Do Valle, and C. Timo-laria, "Studying the interaction between brain structures via directed coherence and Granger causality," *EURASIP Journal on Applied Signal Processing*, vol. 5, no. 1, pp. 40–48, 1998.

[10] L. A. Baccalá and K. Sameshima, "Partial directed coherence: a new concept in neural structure determination," *Biological Cybernetics*, vol. 84, no. 6, pp. 463–474, 2001.

[11] R. Goebel, A. Roebroeck, D.-S. Kim, and E. Formisano, "Investigating directed cortical interactions in time-resolved fMRI data using vector autoregressive modeling and Granger causality mapping," *Magnetic Resonance Imaging*, vol. 21, no. 10, pp. 1251–1261, 2003.

[12] A. Roebroeck, E. Formisano, and R. Goebel, "Mapping directed influence over the brain using Granger causality and fMRI," *NeuroImage*, vol. 25, no. 1, pp. 230–242, 2005.

[13] M. J. Kamiński, M. Ding, W. A. Truccolo, and S. L. Bressler, "Evaluating causal relations in neural systems: Granger causality, directed transfer function and statistical assessment of significance," *Biological Cybernetics*, vol. 85, no. 2, pp. 145–157, 2001.

[14] F. A. Graybill, *Theory and Application of the Linear Model*, Buxbury Press, Boston, Mass, USA, 1976.

[15] H. Lütkepohl, *Introduction to Multiple Time Series Analysis*, Springer, New York, NY, USA, 1993.

[16] P. K. Sen and J. M. Singer, *Large Sample Methods in Statistics - An Introduction with Applications*, Chapman and Hall, New York, NY, USA, 1980.

[17] M. J. Brammer, E. T. Bullmore, A. Simmons, et al., "Generic brain activation mapping in functional magnetic resonance imaging: a nonparametric approach," *Magnetic Resonance Imaging*, vol. 15, no. 7, pp. 763–770, 1997.

[18] J. Talairach and P. Tournoux, *A Co-Planar Stereotaxic Atlas of the Human Brain*, Thieme, Stuttgart, Germany, 1988.

[19] E. R. Kandel, J. H. Schwartz, and T. M. Jessell, *Principles of Neural Science*, McGraw-Hill Medical, New York, NY, USA, 4th edition, 2000.

[20] A. Ikeda, S. Yazawa, T. Kunieda, et al., "Cognitive motor control in human pre-supplementary motor area studied by subdural recording of discrimination/selection-related potentials," *Brain*, vol. 122, no. 5, pp. 915–931, 1999.

[21] M. Humberstone, G. Sawle, S. Clare, et al., "Functional MRI of single motor events," *Annals of Neurology*, vol. 42, no. 4, pp. 632–637, 1997.

**João Ricardo Sato** is a statistician and Ph.D. student. The main areas of his research are time series analysis and neuroimaging. Nowadays, he has been working at the Neuroimaging Laboratory of the University of São Paulo, Brazil.

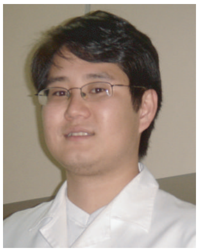

**Daniel Yasumasa Takahashi** is pursuing graduate studies in bioinformatics, Bioinformatics Graduate Program, University of São Paulo, Brazil. His major interest is the application of mathematical techniques to the investigation of interaction between neural populations, which underlies important cognitive tasks.

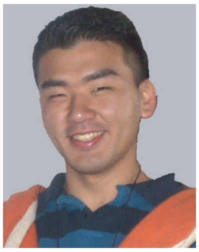

**Ellison Fernando Cardoso** is a medical doctor (radiologist) and Ph.D. student. His work is focused on fMRI studies in patients with Parkinson's disease and major depressive disorder.

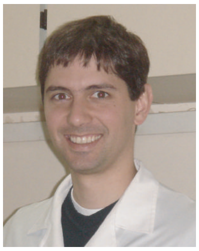

**Maria da Graça Morais Martin** is a medical doctor in Brazil. She graduated from the University of São Paulo Medical School (1992–1997), where she also completed her Radiology Residency (1998–2000). She held a Research Fellowship in fMRI at the University of California San Diego (2004-2005). Her research is focused on fMRI studies, and she is now a Ph.D. student on an fMRI normative database.

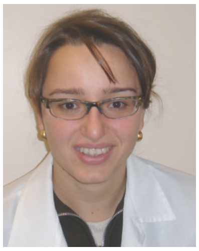

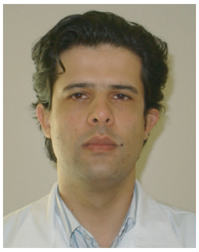

**Edson Amaro Júnior** is an Associate Professor of radiology, and lecturer at the post-graduate program in the Department of Radiology - HCFMUSP and Experimental Neuropsychology - USP. His areas of interest include clinical fMRI, local normative database, DBS in Parkinson's, Parkinson's and depression, ALS and early Alzheimer's disease. Related to these projects he is also supervising a number of doctoral students. Experiment design strategies and methods to deal with the scanner acoustic noise are the main areas of interest. Administrative job includes coordination of the Laboratory for Functional Neuroimaging (NIF), LIM 44, clinical reports in MR and CT scans, and teaching basic MRI to medical students.

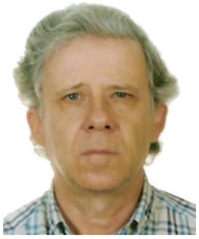

**Pedro Alberto Morettin** received a Ph.D. degree in statistics from the University of California, Berkeley. He is a Professor of statistics at the Department of Statistics, University of São Paulo, Brazil. His interests are in the area of time series analysis and applications to medicine, physical sciences, economics, and finance.

